# Invasive Alien Species as a Potential Source of Phytopharmaceuticals: Phenolic Composition and Antimicrobial and Cytotoxic Activity of *Robinia pseudoacacia* L. Leaf and Flower Extracts

**DOI:** 10.3390/plants12142715

**Published:** 2023-07-21

**Authors:** Mirela Uzelac, Barbara Sladonja, Ivana Šola, Slavica Dudaš, Josipa Bilić, Ibukun M. Famuyide, Lyndy J. McGaw, Jacobus N. Eloff, Maja Mikulic-Petkovsek, Danijela Poljuha

**Affiliations:** 1 Department of Agriculture and Nutrition, Institute of Agriculture and Tourism, Karla Huguesa 8, 52440 Poreč, Croatia; mirela@iptpo.hr (M.U.); barbara@iptpo.hr (B.S.); 2 Department of Biology, Faculty of Science, University of Zagreb, 10000 Zagreb, Croatia; ivana.sola@biol.pmf.hr; 3 Agricultural Department, Polytechnic of Rijeka, Karla Huguesa 6, 52440 Poreč, Croatia; sdudas@veleri.hr; 4 METRIS Research Centre, Istrian University of Applied Sciences, Zagrebačka 30, 52100 Pula, Croatia; jbilic@iv.hr; 5 Phytomedicine Programme, Paraclinical Sciences Department, University of Pretoria, P/Bag X04, Onderstepoort, Pretoria 0110, Gauteng, South Africa; adeyerimi@gmail.com (I.M.F.); lyndy.mcgaw@up.ac.za (L.J.M.); kobus.eloff@up.ac.za (J.N.E.); 6 Department of Agronomy, Biotechnical Faculty, University of Ljubljana, Jamnikarjeva 101, 1000 Ljubljana, Slovenia; maja.mikulic-petkovsek@bf.uni-lj.si

**Keywords:** antioxidant, antibacterial, antibiofilm, antifungal, anti-quorum sensing, black locust, phenolics

## Abstract

Black locust (*Robinia pseudoacacia* L.), an invasive tree in Europe, commonly known for its negative impact on biodiversity, is a rich source of phenolic compounds recognized in traditional medicine. Since the metabolite profile depends on the environment and climate, this study aimed to provide the first LC-MS phytochemical screening of the black locust from the Istria region (Croatia). The compounds were extracted from leaves and flowers with 70% ethanol and 80% methanol. Total phenolics (TP) and flavonoids (TF), as well as antioxidant capacity (AC) measured by ABTS (17.49–146.41 mg TE/g DW), DPPH (24.67–118.49 mg TE/g DW), and FRAP (7.38–77.53 mg TE/g DW) assays, were higher in leaf than in flower extracts. Higher TP and total non-flavonoid (TNF) values were displayed in ethanolic than in methanolic extracts. In total, 64 compounds were identified, of which flavonols (20) and hydroxycinnamic acid derivatives (15) were the most represented. Flavanols such as catechin dominated in leaf extracts, followed by flavonols, with kaempferol glucuronyl rhamnosyl hexosides as the main compound, respectively. Flower extracts had the highest share of flavones, followed by ellagitannins, with luteolin dirhamnosyl hexosides and vescalagin, respectively, being predominant. The extracts had good quorum sensing, biofilm formation prevention, and eradicating capacity. The results provided new insights into the phytochemical properties of *R. pseudoacacia* as the first step toward its potential pharmaceutical use.

## 1. Introduction

The phytochemical composition proved to be very important in the invasion ability of alien plants and, therefore, a leading cause of their environmentally harmful effects [1]. Conversely, this phytochemical richness represents a vast, still underutilized potential for isolating active ingredients and their use as phytopharmaceuticals [2,3]. Plant extracts or specialized metabolites that exhibit specific actions and are non-toxic are being explored as potential pharmaceutical candidates. Furthermore, these compounds could serve as active components in natural, environmentally safer formulations of pesticides and herbicides in organic crop production.

Phenolic compounds, in particular, are an important bioactive group that contribute significantly to the adaptation and growth of invasive alien plant species (IAPS) and protect them from damage and infection [4]. IAPS with a high phenolic compound content may have a competitive advantage over native plants [5]. However, the function and importance of high phenolic diversity are not yet fully comprehended.

*Robinia pseudoacacia* L. (Fabaceae), known as black locust, is a deciduous tree native to North America that was introduced in Europe in the early 17th century as an ornamental tree and later for land reclamation [6]. Today, it is mainly used in the honey-making and wood industries [7]. The black locust is a light-demanding pioneer species that grows in diverse climates and ecological conditions. The species can adapt to prolonged drought by reducing water loss through extenuated transpiration and leaf size [8]. It is a fast-growing species that often inhabits poor soil and anthropogenically disturbed habitats, such as abandoned agricultural land, degraded forest plantations, or polluted urban sites [7,9].

Over 400 years after its introduction, *R. pseudoacacia* is now considered invasive in many European countries, including Croatia [10,11]. This species often overgrows and overshadows indigenous plants, consequently reducing biodiversity [12]. Since the black locust is a nitrogen-fixing plant, it can cause changes in indigenous vegetation towards ruderal and nitrophilous species [13]. Moreover, many studies report the allelopathic potential of this species [14,15,16], which is considered one of the most important mechanisms responsible for the spread of IAPS in new areas [17,18,19]. In particular, these studies highlight the efficacy of whole extracts [20,21] or individual phenolic compounds [14] extracted from different parts of the plant in inhibiting the germination and growth of other plant species.

The opinions of scientists who deal with IAPS are divided between the positive and negative impacts of *R. pseudoacacia* on the economy and the environment [7,22]. Although there are many adverse effects, the positive use of its specialized metabolites is not negligible. A study by Marinas et al. [23] revealed that the phenolics catechin, rutin, resveratrol, and quercetin extracted from the ethanolic extracts of black locust leaves had antimicrobial activity against Gram-positive (*Staphylococcus aureus* Rosenbach, *Bacillus subtilis* Ehrenberg) and Gram-negative (*Pseudomonas aeruginosa* Schröter, *Klebsiella pneumoniae* Schroeter, and *Escherichia coli* Migula) bacterial strains.

Black locust flowers are a rich source of phenolics with proven health benefits, such as epigallocatechin, ferulic acid, hyperoside, and rutin (also known as rutoside, quercetin-3-rutinoside, and soforin) [24,25]. Epigallocatechin is known for its antioxidant, anti-inflammatory, and anti-apoptotic properties in the treatment of various kidney diseases [26], while ferulic acid is widely used in skin care formulations as a photoprotectant, melanogenesis inhibitor, and wound healing accelerator [27]. The compound hyperoside has promising anticancer activity against different cancer types, such as lung, cervical, colon, and breast cancer, as well as anti-inflammatory, antibacterial, antiviral, antidepressant, and cardioprotective activity [28]. The flavonoid rutin is known for its efficiency in treating neurodegenerative diseases [29]. The flowers of *R. pseudoacacia* are also a source of luteolin, gallic, and caffeic acids, which are known as antioxidant, anti-inflammatory, and antimicrobial agents [30,31,32]. Black locust in traditional medicine, especially in Asia, is used as a diuretic, anti-inflammatory, spasmolytic, and sedative due to its rich content of specialized metabolites [33].

Lately, there has been a growing interest in exploring the impact of substances on bacterial biofilms, alongside the conventional testing of antibacterial efficacy. Biofilms formed by pathogenic microbes make them more resistant to the action of antimicrobials than their platonic counterparts. This is because, in the biofilm mode, microbes are protected by a network that is composed of various organic and non-organic components that house the pathogens and promote strong attachments to various surfaces. Within this matrix, pathogens, in mono- or polycultures, can also transfer resistant genes among themselves, making them more antimicrobially resistant [34]. However, to form biofilms, microbes must communicate with each other via specific biosignals. This molecular communication, which influences the biofilm behavior of microbes, is called quorum sensing. Apart from biofilms, quorum sensing also regulates the surface motility and pathogenicity of microbes [35]. Therefore, inhibiting quorum sensing (quorum quenching) is an alternative antimicrobial strategy. Several studies have reported the potential antibiofilm and antiquorum activities of natural botanical products [36,37,38].

The production and accumulation of a plant’s specialized metabolites are influenced by various ecological factors, such as temperature, soil salinity and fertility, light, carbon dioxide, water availability, herbivore or pathogen infestation, competition from neighboring plants, and different developmental stages [4]. For example, under abiotic stress conditions, the production of specialized metabolites can vary by up to 50% [39]. Hence, it is crucial to explore the specialized metabolites of plants in a location- and organ-specific manner. Understanding the unique phytochemical and phenolic profiles of non-native invasive plant species in the regions where they spread is essential, given that plant invasion is associated with high levels of biotic and abiotic stress [40]. This knowledge is also a fundamental requirement for the effective management of invasive species and their potential utilization as ecosystem service providers. In situations where IAPS such as *R. pseudoacacia* have already extensively spread and eradicating them is no longer a feasible management approach, exploiting the ecosystem service potential of IAPS could establish a foundation for alternative sustainable management strategies. By gaining new insights into the bioactive compounds they contain, there could be increased motivation to monitor existing populations more rigorously and to remove them from natural environments.

This research aims to contribute to that goal from the perspective of Croatian black locust populations. Namely, so far, there have been no data on the phytochemical profile of black locust, except for its honey products [41,42,43]. Thus, the objective of this study is to assess the phytopharmaceutical potential of leaf and flower extracts of *R. pseudoacacia* from Istria (Croatia) in different solvents by a combination of spectrophotometric, chromatographic, cell culture, and chemometric analyses. For this purpose, we (a) spectrophotometrically measured the content of different groups of bioactive compounds (total phenolics, flavonoids, and non-flavonoids) and their antioxidant capacity using three standard assays; (b) identified and quantified the main phenolic compounds using the LC-DAD-MS method; (c) tested the antibacterial, antifungal, and cytotoxic activities using a two-fold microdilution assay; (d) evaluated the anti-biofilm and anti-quorum activities; and (e) statistically determined the relationships among the samples and the measured parameters using one-way ANOVA, Tukey’s test (*p* ≤ 0.05 and 0.01), principal component analysis, and Pearson’s correlation coefficient. In this study, we also show how the choice of solvent and plant organ affected the measured variables and discuss the influence of the site-specific environment on the phenolic profile.

This study is the first to present a comprehensive LC-DAD-MS polyphenolic profile of the flowers and leaves of *R. pseudoacacia* in Croatia, along with an evaluation of their biological activity. Furthermore, it serves as a starting point for developing a sustainable management model for this invasive species in the area, which can potentially be implemented in a broader geographical region.

## 2. Results and Discussion

### 2.1. Spectrophotometric Analysis of the Phenolic Content and Antioxidant Capacity

**Figure 1:**
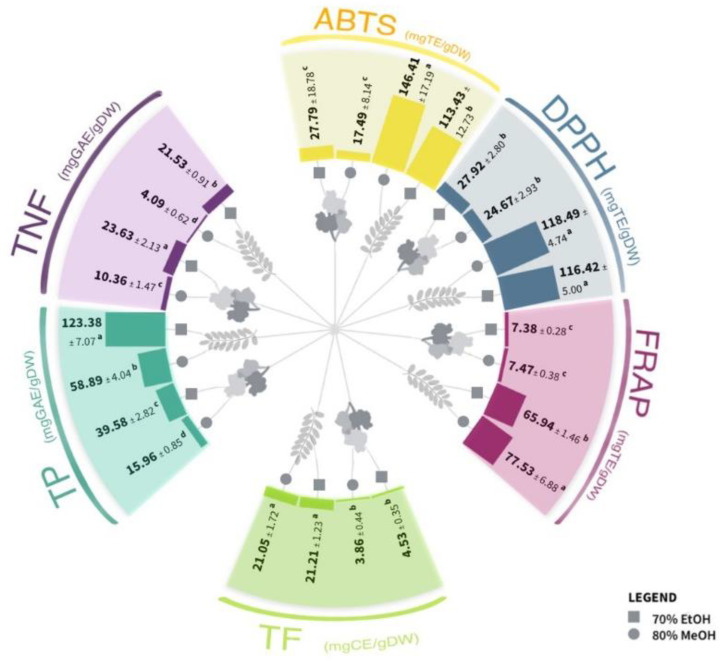
The content of total phenolics (TP), total non-flavonoids (TNF), and total flavonoids (TF) and antioxidant capacity (obtained by DPPH (2,2-diphenyl-2-picrylhydrazyl) free radical assay, ABTS [2,2′-azino-bis (3-ethylbenzothiazoline-6-sulphonic acid)] radical cation assay, and FRAP (ferric reducing antioxidant power) assay) in *Robinia pseudoacacia* leaf and flower extracts in two solvents. Different letters (a–d) in the same section indicate significant differences between the measured values (two-way ANOVA, Tukey’s test, *p* ≤ 0.01). The results are expressed in gallic acid equivalents per g of dry weight (mg GAE/gDW) for TP and TNF and for TF in catechin equivalents per g of dry weight (mg CE/gDW).

Spectrophotometrically determined total phenolic (TP) and total flavonoid (TF) contents, as well as antioxidant capacity (AC), were higher in leaf than in flower extracts. Higher values of TP and TNF were displayed in ethanolic extracts compared to methanolic extracts. The highest values of both TP and TF content were obtained in ethanolic extracts of leaf samples (123.38 mg of gallic acid equivalent (GAE)/g of dry weight (DW) and 21.21 mg of catechin equivalent (CE/g of dry weight (DW), respectively), while ethanolic flower extract expressed the highest total non-flavonoid (TNF) content (23.63 mg GAE/g DW) (Figure 1). Since only about 10% of the medicinal plant material has a concentration of total phenolics higher than 5% DW of GAE [44,45], both ethanolic and methanolic leaf extracts are among the plant materials with the highest concentration of these compounds.

Our tested extracts displayed equivalent or higher levels of measured concentrations of total phenolics compared to the concentrations of total phenolics found in ethanolic and methanolic extracts of black locust leaves and flowers in previous studies [23,46,47]. The data obtained indicate that black locust populations from Istria represent a valuable source of total phenolics and should be taken into account when considering this species as a potential biomaterial for phytopharmacy and, possibly, the food industry. Recently, a few studies explored the nutrient composition and content of bioactive compounds in edible flowers, including black locust inflorescences [46,48], referring to them as a potentially valuable food additive due to their high biological value. This is also confirmed by the increasing trend of using black locust flowers in the preparation of various food items, such as salads, pastries, jams, honey, and syrups [48,49].

In our study, the antioxidant capacity of extracts was assessed by three standard methods: the DPPH (2,2-diphenyl-2-picrylhydrazyl) free radical assay, the ABTS [2,2′-azino-bis (3-ethylbenzothiazoline-6-sulphonic acid)] radical cation assay, and the FRAP (ferric reducing antioxidant power) assay. All applied assays revealed a higher antioxidant capacity of the methanolic than ethanolic leaf extracts, while no statistically significant differences between solvents were observed in flower extracts (Figure 1). These results were expected since it is known that extraction in polar solvents (such as methanol and ethanol), in contrast to non-polar solvents (e.g., hexane), contains a higher amount of total phenolic content with strong reducing power and radical scavenging activity [50]. Thus, our study focused on selecting appropriate polar solvents, considering that ethanol is a safe solvent for human consumption and is known to be a good solvent for polyphenolic extraction. On the other hand, methanol is typically more effective in extracting polyphenolics with lower molecular weights [51].

**Figure 2:**
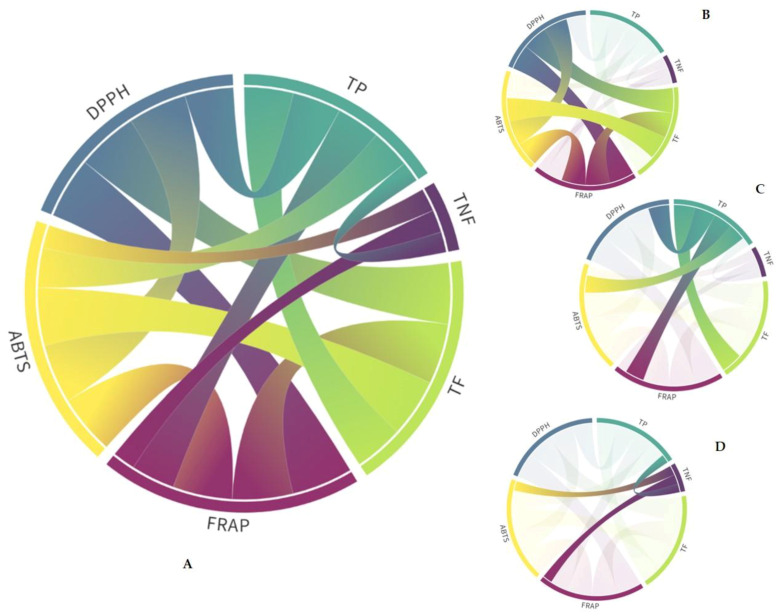
Pearson’s correlation coefficients of total phenolics (TP), non-flavonoids (TNF), and flavonoids (TF) contents and antioxidant capacities determined by ABTS [2,2′-azino-bis (3-ethylbenzothiazoline-6-sulphonic acid)] radical cation assay, DPPH (2,2-diphenyl-2-picrylhydrazyl) free radical assay, and FRAP (ferric reducing antioxidant power) assays. (**A**) Only significant correlations (*p* ≤ 0.05; *p* ≤ 0.01; Table S1) are shown. (**B**) Total correlations (0.9 < r < 1.0). (**C**) Very strong and strong correlations (0.50 < r < 0.9). (**D**) Weak correlations (0.25 < r < 0.4).

In all extracts, a highly significant (*p* ≤ 0.01) total positive correlation (0.9 < r < 1.0) was observed between TF content and AC values determined by ABTS, DPPH, and FRAP assays (Table S1, Figure 2A,B), as well as between the three AC assays. In addition, a very strong (0.75 < r < 0.9) or strong (0.50 < r < 0.75) positive correlation according to the Roemer–Orphal scale between TP content and AC values determined by all applied assays was observed (Table S1, Figure 2A,C), in agreement with the results of Ji et al. [52]. The flavonoids quercetin and kaempferol and their derivates possess the ability to reduce ROS (reactive oxygen species) in plants during stress conditions [53].

Weak (0.25 < r < 0.4) positive correlations were observed between TNF and TP (*p* ≤ 0.05) and weak negative correlations between TNF and AC (ABTS and FRAP), as shown in Table S1 and Figure 2A,D. Negative correlations suggest that some of the TNF might have prooxidative activity.

### 2.2. Phenolic Compounds

The majority of research on the phytochemical composition of *R. pseudoacacia* has focused on its wood [54,55,56] and bark [24] as a source of biomass for bioenergy and fuels. Furthermore, there has been a significant investigation into the chemical properties of black locust honey [57,58,59,60]. The leaves and flowers of *R. pseudoacacia* are a rich source of phenolic compounds, especially flavonoids [25,61,62], with proven bioactivity and health benefits [48,56]. The leaves and flowers of the invasive *R. pseudoacacia* could serve as a free, renewable source of bioactive phenolic compounds that could be used in the pharmaceutical industry. However, specific environmental conditions resulting from different geographical locations, such as different average temperatures, irradiation, rainfall, and heavy metal contamination [63,64], affect specific metabolites and can significantly alter the phytochemical profile of a species. Therefore, a thorough phytochemical analysis of plant populations from different geographical areas is a prerequisite for a reliable statement about their biopotential. Identification and quantification of chemical compounds in black locust leaves and flowers can improve understanding of the underlying mechanisms of their bioactivity and potentially contribute to understanding invasion mechanisms. In our broader study area, there are no studies on the phenolic composition of the black locust plant. Our study is the first to present a comprehensive LC-DAD-MS phenolic profile of *R. pseudoacacia* leaves and flowers in Croatia.

**Figure 3:**
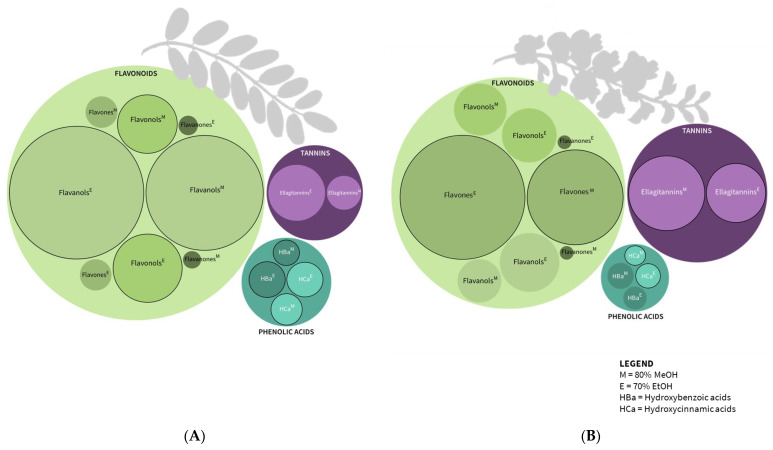
The main phenolic groups obtained by the LC-DAD-MS method in the (**A**) leaf and (**B**) flower extracts. The ratio of the circles’ sizes corresponds to the total concentration of individual phenolic groups (mg/g of dry weight (DW)). The circle border indicates statistically significant differences between concentrations of individual phenolic groups within the plant organ (ANOVA, Tukey’s test, *p* ≤ 0.01).

We identified, in total, 64 distinct phenolic compounds, 60 in leaf and 55 in flower extracts (Table 1). The identified compounds belong to the groups of phenolic acids (hydroxycinnamic and hydroxybenzoic acid derivatives), flavonoids (flavones, flavanols, flavonols, and flavanones), and non-flavonoids (ellagitannins) (Table 1, Figure 3). The ethanolic leaf extract displayed the highest concentration of total identified compounds (52.72 mg/g DW), followed by the methanolic leaf extract, with a recorded 36.74 mg/g DW of total phenolics. Utilizing methanol as a solvent during the flower extract preparation yielded a greater concentration of total identified phenolic compounds (32.74 mg/g DW) compared to ethanol (24.85 mg/g DW). For all phenolic groups, both the organ and the solvent had a significant effect on the total concentration, except for flavanones, where only the plant organ had an influence (two-way ANOVA and Tukey’s test at *p*-value ≤ 0.01) (Table 1).

The suitability of different solvents for the extraction of phenolics from different parts of the plant (70% ethanol for leaves and 80% methanol for flowers) suggests that the concentration of phenolic components in *R. pseudoacacia* leaf and flower is different. In addition, other components might also differ and consequently affect the extraction of phenolic compounds synergistically or antagonistically [65]. Polyphenolics are compounds with high solubility in polar solvents [66]. In particular, methanol has been generally found to be more efficient in the extraction of lower molecular weight polyphenolics, while higher molecular weight flavanols are better extracted with aqueous acetone [67,68,69,70]. In the study of Gajik et al. [25], the authors analyzed the influence of ethanol concentration and extraction conditions on the total content of phenolics extracted from the black locust flowers and determined 60% ethanol (*v*/*v*) as the optimal concentration of ethanol as a solvent. In our research, we used a slightly higher ethanol percentage as it was found to be the most effective in our previous study [3,71].

In the leaf extracts, the flavanols dominated, accounting for 62% of the total analyzed phenolics (TAP) in EtOH and 66% TAP in MeOH extracts, followed by the flavonols (16% TAP in both solvents) (Figure 3A, Table 1). Flower extracts had the highest share of flavones (46% TAP in EtOH and 59% TAP in MeOH), followed by ellagitannins with a share of 16% TAP in EtOH and 21% TAP in MeOH extracts (Figure 3B, Table 1). In the study of Veitch et al. [61], carried out on the specimens from the living collections at the Royal Botanic Gardens, Kew (UK), scientists compared the flavonoid chemistry of black locust leaves and flowers. Their research demonstrated that the leaves of *R. pseudoacacia* contained a higher concentration of flavone 7-*O*-glycosides, specifically acacetin, while the flowers were found to have more flavonol 3,7-di-*O*-glycosides. The glycosylation patterns of the compounds revealed tissue-specific variations in flavonoid chemistry, as demonstrated by their research findings [61].

As expected, the highest number of individual compounds were identified in the groups of flavonols (20), hydroxycinnamic acids (15), and flavanols (14) (Table 1). This can be attributed to the proven prevalence of flavonoids, alkaloids, terpenoids, tannins, and phenolics, such as kaempferol, quercetin, and phenolic acids, as well as their derivatives, in the Fabaceae family [72].

The compounds detected in all extracts in the highest concentrations were catechin, procyanidin, luteolin, apigenin, quercetin, kaempferol, isorhamnetin, vescalagin, and hydroxybenzoic acid derivatives, with catechin (6.22 mg/g DW) and kaemferol glucuronyl rhamnosyl hexoside 2 (5.52 mg/g DW) as dominant in leaf extracts and luteolin dirhamnosyl hexoside 1 (12.30 mg/g DW) and vescalagin 1 (5.86 mg/g DW) in flower extracts (Table 1). A high concentration of flavone glycosides (luteolin, apigenin, kaempferol, and catechins) detected in our study has already been found in other plant species inhabiting their invasive range [40]. Quercetin and kaempferol were reported to act as allelopathic agents in other plant species [40,73,74,75,76]. Further studies are needed to determine whether these compounds may have an effect on the invasive ability.

Nine compounds were leaf-specific (*p*-coumaric acid, ferulic acid, sinapic acid derivate, *p*-hydroxybenzoic acid, procyanidin trimers 4 and 5, procyanidin tetramers 1 and 2, and kaempferol-3-glucoside), and four were flower-specific (quercetin pentoside, kaempferol hexosides 1, 2, and 3) (Table 1). This suggests the organ-specific function(s) of the abovementioned compounds and the importance of quercetin pentoside and kampferol hexosides in the generative organs of black locust. The representation of certain phenolic compounds in a particular organ of the plant has already been recorded [77].

Phenolic compounds identified in our study, whose main role may be plant protection, have been found to have antioxidant, antimicrobial, cytoprotective, anticarcinogenic, neuroprotective, and cardioprotective activities [78,79]. Catechin is a naturally occurring flavanol originally derived from catechu, the tannic juice or boiled extract of *Acacia catechu* L. (Fabaceae) [80]. This compound shows strong antioxidant capacity due to its redox potential. Furthermore, catechin is known for its anti-inflammatory activity and as a protective compound against cardiovascular diseases and cancer [81,82,83]. The study of Boskov et al. [84] revealed a high concentration of catechin hydrate in black locust flower aqueous extract (832 mg/100 g DW) and (12 mg/100 g DW) in the 50% (*v*/*v*) methanol extract. Marinas et al. [23] recorded catechin (0.9 µg/mL) in the black locust leaf and epicatechin (0.24 µg/mL) and catechin (0.13 µg/mL) in the 70% ethanol seed extract of *R. pseudoacacia*. Kaempferol and some of its glycosides exert strong antioxidant activity both in vitro and in vivo [85]. The study of Veitch et al. [61] analyzed several kaempferol derivates in black locust flowers, as did Kulevanova et al. [86], who found a high concentration of kaempferol (0.892 ω/%). Loizzo et al. [47] investigated the black locust flowers to assess their potential as functional foods. Quercetin-3-rutinoside, also called rutin, was the most representative flavonoid (28 mg/g DW), followed by kaempferol (2.4 mg/g DW). Rutin is a flavonol abundant in plants and known for its antioxidant, cytoprotective, anticarcinogenic, neuroprotective, and cardioprotective activities [87,88,89]. Rutin was found by Savić-Gajić et al. [25] in ethanolic black locust flower extract (57 mg/100 g DW). In the study of Nasir et al. [14], the compounds quercetin, robinetin, and myricetin were responsible for allelopathic suppression of the root and shoot growth of lettuce. In our research, the highest concentration of phenolics (12.30 mg/g DW) was detected for luteolin derivates in the flower extracts. Luteolin is a flavonoid found in different plants, such as vegetables, medicinal herbs, and fruits. Luteolin exhibits multiple biological effects, such as anti-inflammation, anti-allergy, and anticancer [30]. This compound, derived from a black locust flower, suggests its potential as an insecticide for Lepidoptera larvae [90]. Finally, a significant amount of vescalagin was found in both the leaf (3.30 mg/g DW) and flower (5.86 mg/g DW) extracts in this study. Vescalagin is a compound classified as ellagitannin that has proven antibacterial activity against *Staphylococcus epidermidis*, *Staphylococcus aureus*, and *Pseudomonas aeruginosa* [79].

### 2.3. Principal Component Analysis of Specialized Metabolites and Antioxidant Capacity

According to ANOVA analysis for all phenolic groups (Table 1), organs had a significant effect on the total concentration: the only exceptions were hydroxybenzoic acid derivatives in methanolic extracts and ellagitannins in ethanolic extracts (two-way ANOVA and Tukey’s test at *p*-value ≤ 0.01).

**Figure 4:**
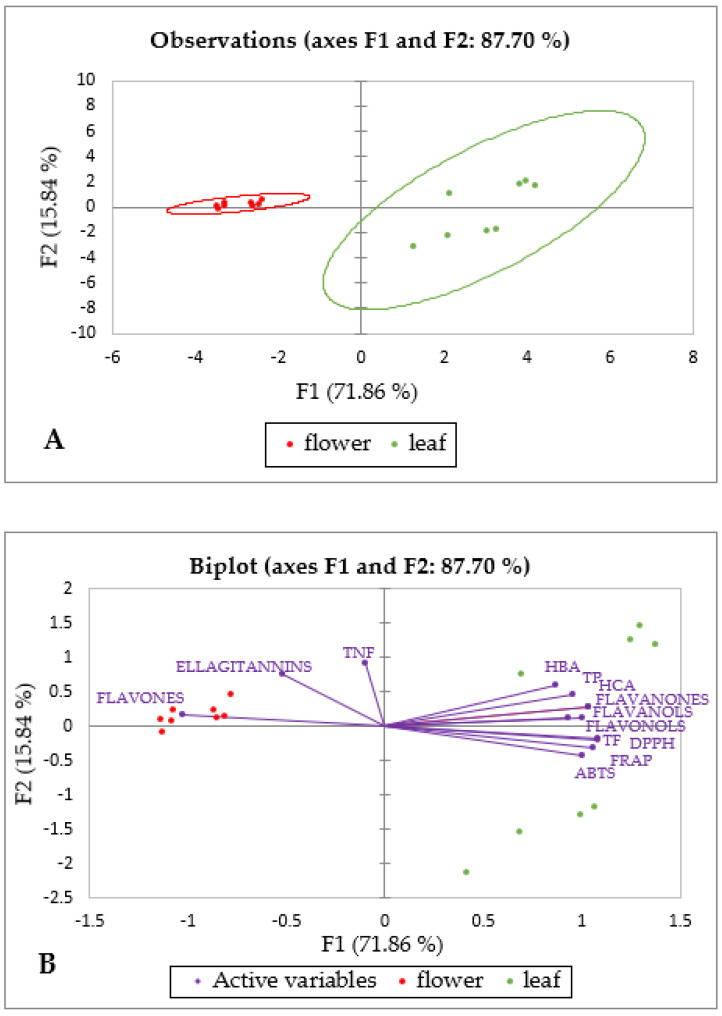
Principal component analysis (PCA) of the groups of metabolites and antioxidant capacity of *Robinia pseudoacacia* leaf and flower extracts in two solvents (70% EtOH and 80% MeOH). (**A**) Score plot separating the flower and leaf extracts based on the measured groups of metabolites and antioxidant capacity; (**B**) loading plot of the measured variables; TP = total phenolics, TF = total flavonoids, TNF = total nonflavonoids, ABTS = 2,2′-azino-bis (3-ethylbenzothiazoline-6-sulfonic acid) diammonium salt assay, FRAP = ferric reducing antioxidant power assay, DPPH = 2,2-diphenyl-1-picrylhydrazyl assay, HBA = Hydroxybenzoic acids, HCA = Hydroxycinnamic acids. The differences between different populations are not reported in this contribution.

For the phenolic group of hydroxycinnamic acid derivatives, both the organ and the solvent had a significant effect on the total concentration. The principal component analysis (PCA) based on the measured groups of metabolites (Table 1, Figure 1) and antioxidant capacity (Figure 1) explained 87.7% of the total variation among the samples, where PC1 accounted for 71.86% of the variance and PC2 for 15.84% (Figure 4A). Leaf and flower extracts were clearly separated based on the measured groups of metabolites and antioxidant capacity, confirming the results of the ANOVA analysis (Table 1). PCA revealed that flavones, ellagitannins, and total non-flavonoid compounds contributed mostly to the separation of flowers, while all the other groups of analyzed metabolites contributed to the separation of leaf extracts (Figure 4B). Interestingly, both groups of phenolic acids as well as antioxidant capacity measured by three different methods clearly predominated in the extracts of leaves. Since the flowers are present for only a short time, about two weeks [21,91], and the leaves much longer, we assume that a higher concentration of phenolic antioxidants in the leaves is a likely condition for their longer shelf life. A similar result was found in the flowers and leaves of Tunisian cultivars of *Punica granatum* [92] and *Elaeagnus angustifolia* [93].

### 2.4. Antimicrobial and Cytotoxic Activity

#### 2.4.1. Antibacterial and Antifungal Activity and Cytotoxicity

The antibacterial and antifungal activities of the ethanol and methanol extracts of the two tested samples are given in Table 2, respectively. The antimicrobial activity of plant extracts was classified based on the MIC values as follows: good (<0.1 mg/mL), moderate (0.1–0.63 mg/mL), and weak (>0.63 mg/mL) [94]. Eloff [95] investigated the antimicrobial activity of acetone leaf extracts from 537 South African tree species against four nosocomial bacteria and two yeasts. He proposed the following categories based on MICs in mg/mL: outstanding activity (0.02 mg/mL and lower), excellent activity (0.021–0.024), very good activity (0.04–0.08), good activity (0.081–0.16), average activity (0.161–0.32), and weak activity (>0.32). These values were based on the top 1, 3, 9, 25, and 50% of all activities. Based on this proposal, the samples tested had weak antibacterial and antifungal activity against the tested pathogens, but the focus of this contribution was on phenolics. If acetone were used as an extractant, much better activity would be found [96]. Leaf extracts exhibited higher antimicrobial activity than flower extracts. The value of total antibacterial activity (TAA), expressed in mL/g for each sample tested, was determined by dividing the yield of each extract by the respective MIC values. This shows the largest volume to which the biologically active compounds present in 1 g of the extract can be diluted and still inhibit the growth of the test organism [97]. This value gives an idea of the efficacy of the plant extract. In this study, the methanol extract of *R. pseudoacacia* flower had the best TAA value of 403, respectively, against *Pseudomonas aeruginosa* and *Bacillus cereus*, while the methanol leaf extract of *R. pseudoacacia* had the best TAA value of 392, respectively, against *Candida albicans* and *Candida parapsilosis* (Table 2). In essence, a TAA value of 392, for example, means that antibacterial activity can still be observed when 1 g of *R. pseudoacacia* methanol extract is diluted 392 times.

The cytotoxicity was determined using an in vitro assay against African green monkey (*Cercopithecus aethiops* L.) kidney cells (Vero cells). The LC_50_ and the selectivity index (SI) values were calculated (Table 2). Apart from determining the minimum inhibitory potential of plant extracts, it is equally important to note their potential toxicity in mammalian cells. In classifying the in vitro cytotoxicity of plant extracts, values below 20 µg/mL are considered highly cytotoxic [94]. This study shows all the tested extracts had low toxicity against Vero cells (Table 2). The LC_50_ values of the flower extracts, in particular, were above 1 mg/mL. In vitro cytotoxicity determination, however, should be followed up with in vivo experiments in order to ascertain the safety of the extracts in a whole organism. This is because in vitro data may not always agree with in vivo studies due to pharmacokinetic factors and gut interactions [98].

#### 2.4.2. Anti-Biofilm and Anti-Quorum Activities

Table 3 shows the antibiofilm and anti-quorum activity of the tested extracts at a single concentration of 1 mg/mL against *Staphylococcus epidermidis* (ATCC 35984). This bacterium, a known high biofilm former, was previously isolated from a patient with intravascular catheter-associated sepsis [99]. It has been used as a model in many antibiofilm studies. All the tested samples had the capacity to either prevent or destroy the formed biofilms. The *R. pseudoacacia* leaf and flower methanol extracts had good biofilm prevention activity. The antibiofilm action of these extracts may be due to their potential interference with biofilm-generating and/or perpetuating factors. For example, they may have modulated the production of adhesins such as the polysaccharide intercellular adhesin (PIA) encoded by the intercellular adhesion (ica) operon in *Staphylococcus epidermidis* [100].

Further experiments are essential to substantiate this. In another view, the extracts may have depleted some essential nutrients needed for biofilm formation. Furthermore, the extract may have also interfered with the communication (quorum sensing) signals activating biofilm formation in the bacteria. Table 3 showed that the extracts had anti-quorum sensing activity against *Chromobacterium violaceum* (ATCC 12472), with the *R. pseudoacacia* flower methanol extract having the best minimum quorum sensing inhibitory concentration of 0.16 mg/mL, while the same plant extract and its ethanol counterpart had the best half maximal inhibitory activity of 0.04 mg/mL, respectively. Our study corroborates previous reports showing that plant extracts possess antibiofilm and anti-quorum properties [101,102,103,104].

## 3. Materials and Methods

### 3.1. Chemicals

All chemicals used for extraction and analysis of TP, TNF, and TF were HPLC graded and obtained from Sigma-Aldrich (St. Louis, MI, USA). For spectrofotometrical analysis of antioxidant activity, DPPH (2,2-diphenyl picryhydrazyl), ABTS (2,20-azino-bis(3-ethylbenzothiazoline-6-sulfonic acid), and FRAP (ferric-2,4,6-tripyridyl-D-triazine) complexes were used.

The external standards used in LC-MS assays were: caffeic acid, apigenin-7-glucoside, ferulic acid, quercetin-3-*O*-rhamnoside, neochlorogenic (3-caffeoylquinic) acid, naringenin, ellagic acid, gallic acid, chlorogenic acid, and rutin (quercetin-3-*O*-rutinoside) from Sigma-Aldrich (St. Louis, MI, USA) Chemie; (-)epicatechin, quercetin-3-*O*-galactoside, quercetin-3-*O*-glucoside, *p*-coumaric acid, procyanidin B1, and kaempferol-*O*-glucoside from Fluka Chemie; quercetin-3-*O*-xyloside and quercetin-3-*O*-arabinopyranoside from Apin Chemicals (Abingdon, UK); and isorhamnetin-3-*O*-glucoside from Extrasynthese (Genay, France).

### 3.2. Plant Material

**Figure 5:**
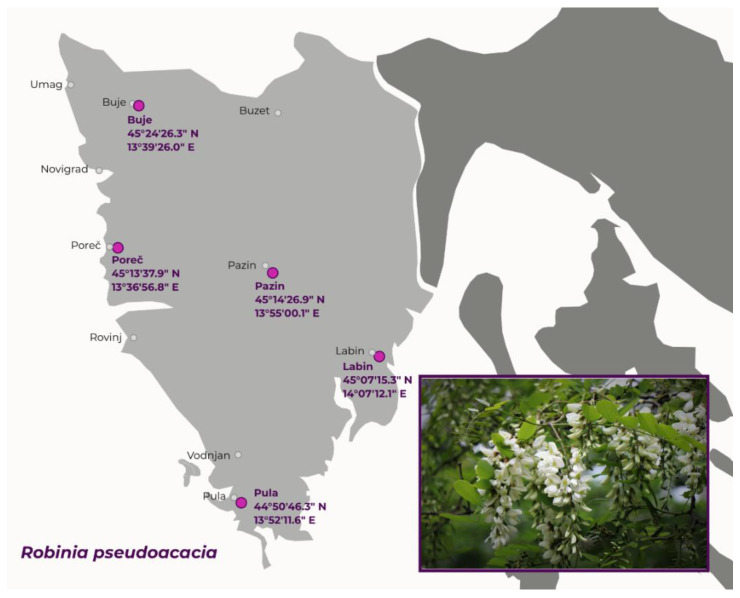
The locations of collected leaf and flower samples of *R. pseudoacacia.* Purple dots indicate groups of locations.

The leaves and inflorescences of *R. pseudoacacia* were collected during the vegetation year 2021, from June to September, in the Istria region (Croatia). The 15 samples were collected in five groups of locations. The groups of sampling locations are presented in Figure 5. After harvesting, the plant material was air-dried in the shade at room temperature. Before grinding the samples for extraction, we separated the individual flowers from the inflorescences and therefore use the term “flower” extracts throughout the paper.

### 3.3. Extraction Procedure

Approximately 250 g of dry plant material from every location was pooled to obtain a representative sample for the research area. The material was finely minced using a Grindomix GM 200 knife mill (Retsch, Haan, Germany) programmed at 10,000 rpm/30 s. For the spectrophotometrical analyses, extracts were prepared in quadruplicate in three repeats (n = 12), according to standard protocol [105]. In the tubes, 0.06 g of plant material was dissolved in 2 mL of solvent (70% EtOH and 80% MeOH). The prepared suspensions were shaken on a shaker-incubator (lES-20, Biosan Medical-Biological Research & Technologies Company, Latvia) at room temperature for 75 min, followed by centrifugation at 12,000 rpm/1 min (Jouan MR23i, Jouan S.A., Saint-Herblain, France) and filtration through 0.20 µm polytetrafluoroethylene (PTFE) filters (Macherey-Nagel, Düren, Germany) and stored at +4 °C until analysis.

The extraction of phenolic compounds for HPLC–DAD-MS identification was performed using the protocol of Mikulic Petkovsek et al. [106]. Briefly, two hundred milligrams of dried plant tissue was extracted with 6 mL of 70% EtOH or 80% MeOH containing 3% (*v*/*v*) formic acid in a cooled ultrasonic bath at (600 W) for 60 min. Extracts were centrifuged for 10 min at 10,000× *g* and filtered through 0.2 µm polytetrafluoroethylene (PTFE) filters (Macherey-Nagel, Düren, Germany).

For bioassays (cytotoxicity and antimicrobial activities), besides MeOH (80%) and EtOH (70%), absolute acetone was additionally used as a solvent for powder extraction. Acetone has the additional advantage of high efficiency in extracting compounds with a wide range of polarities [107]. Briefly, 1 g of ground dried samples was extracted with 10 mL of solvent (ratio 1:10). The mixture was sonicated in a bath (EUMAX^®^ Ultrasonicator, South Africa) for 20 min, and centrifuged at 4000× *g* for 10 min (Hettich Centrifuge, Rotofix 32 A, Labotec, Johannesburg, South Africa). The supernatant was collected, filtered through Whatman No. 1 filter paper into pre-weighed glass vials, and completely dried under a stream of cold air. The dried extracts were weighed, and the yield was obtained by dividing the mass extracted by the initial mass.

### 3.4. Total Phenolics, Flavonoids, and Non-Flavonoids Content and Antioxidant Capacity

The determination of total phenols and phenolic subgroups, along with different antioxidant capacity assays, were measured spectrophotometrically for plant leaf and flower extracts in two solvents (70% EtOH and 80% MeOH) using different methods adjusted to a 2 mL cuvette volume. All measurements were performed in triplicate using a NanoPhotometer P300 spectrophotometer (Implen GmbH, München, Germany).

TP was determined using the method of [108], while TNF was measured using the method described by Amerine et al. [109]. Both methods are based on the reduction of the Folin–Ciocalteu reagent in the presence of phenolics, leading to the formation of molybdenum–tungsten blue that is measured spectrophotometrically at 765 nm. Extract samples for TP (0.02 mL) were diluted with 1.58 mL of distilled water, 0.1 mL of Folin–Ciocalteu reagent (diluted with distilled water 2:1 right before use), and 0.3 mL of a 20% sodium carbonate solution. The samples were incubated for 2 h, and the absorbance was measured spectrophotometrically at 765 nm. For TNF analysis, extracts (0.06 mL) were mixed with 0.03 mL HCl (1:4) and 0.03 mL formaldehyde and left overnight to precipitate. The next day, from the prepared solution, 0.02 mL of the sample was diluted with 1.58 mL of distilled water, 0.1 mL Folin–Ciocalteu reagent, and 0.3 mL 20% sodium carbonate solution. The samples were incubated for 2 h, and the absorbance was measured spectrophotometrically at 765 nm. The results were calculated according to the calibration curve for gallic acid (y = 0.0004x, y = absorbance at 765 nm, x = concentration of gallic acid mg/L, R^2^ = 0.9915).

TF content was measured following the protocol described by Martins et al. [110]. The samples (0.02 mL) were diluted with 0.88 mL of distilled water, 0.06 mL of 5% sodium nitrite, 0.06 mL of 10% aluminum chloride, and 0.8 mL of 4% sodium hydroxide. The samples were incubated for 15 min, and the absorbance was measured spectrophotometrically at 510 nm. The results of TF were calculated according to the calibration curve for gallic acid (y = 0.0004x, y = absorbance at 765 nm, x = concentration of gallic acid mg/L, R^2^ = 0.9915). The results of TNF were calculated according to the calibration curve for catechin (y = 0.0024x, y = absorbance at 510 nm, x = concentration of catechin mg/L, R^2^ = 0.9933). The results of TP and TNF were expressed as mg of gallic acid equivalents (GAE), while TF was expressed as mg of (+)-catechin equivalents (CE) per g of plant dry weight (DW).

The antioxidant capacity (AC) of the extracts was determined spectrophotometrically using standard DPPH, ABTS, and FRAP assays, according to the procedure described in detail in [71]. The results were expressed as mg of Trolox equivalents (TE) per g of DW. The results of DPPH were calculated according to the calibration curve for TROLOX (y = 44.911x, y = absorbance at 517 nm, x = concentration of TROLOX mmol/L, R^2^ = 0.9772). The results of ABTS were calculated according to the calibration curve for TROLOX (y = 46.137x, y = absorbance at 734 nm, x = concentration of TROLOX mmol/L, R^2^ = 0.9811). The results of FRAP were calculated according to the calibration curve for TROLOX (y = 1.638x, y = absorbance at 593 nm, x = concentration of TROLOX mmol/L, R^2^ = 0.9926).

### 3.5. HPLC-DAD-MS Analysis and Identification of Phenolic Compounds

Each MeOH and EtOH extract was subject to detailed LC-DAD-MS analysis to identify and quantify particular phenolic compounds. The separation of the analytes was carried out using HPLC (Dionex UltiMate 3000, Thermo Fisher Scientific, San Jose, CA, USA) with a DAD detector at a column temperature (Gemini C18, Phenomenex, Carlsbad, CA, USA) of 25 °C. Compounds were recorded at wavelengths of 280 and 350 nm. Two mobile phases were used for the separation of phenolic compounds: for mobile phase A, we used double distilled water/acetonitrile/formic acid (96.9/3/0.1, *v/v/v*); and for mobile phase B, we used acetonitrile/double distilled water/formic acid (96.9/3/0.1, *v/v/v*). Samples were eluted according to a linear gradient from 5% to 20% B in the first 15 min, followed by a linear gradient from 20% to 30% B for 5 min, then an isocratic mixture for 5 min, followed by a linear gradient from 30% to 90% B for 5 min, and then an isocratic mixture for 15 min before returning to the initial conditions according to the method described by Mikulic Petkovsek et al. [111]. The volume of injected samples was 20 μL, and the mobile phase flow rate was 0.6 mL/min. Individual metabolites were identified by mass spectrometry (LTQ XL Linear Ion Trap Mass Spectrometer, Thermo Fisher Scientific, San Jose, CA, USA) with electrospray ionization (ESI) in negative scanning under the modified parameters given by Mikulic Petkovsek et al. [112]. The scanning range was from *m/z* 110 to 1700—a data-dependent full scan. The phenolic compounds were confirmed based on the fragmentation products, comparison of the retention times of the corresponding standards, and comparison of the spectra of the individual peaks with the standards. The contents of phenolic compounds were calculated from the peak areas of the samples and the corresponding standard curves of the phenolic compounds. The contents of phenolic compounds in invasive species were expressed in mg/g DW.

### 3.6. Antibacterial and Antifungal Activity

The bacterial isolates were *Pseudomonas aeruginosa* (ATCC 27853), *Staphylococcus aureus* (ATCC 29213), and *Bacillus cereus* (ATCC 21366), while the fungal isolates were *Candida albicans* (C.-P.Robin) Berkhout (ATCC 10231) and *Candida parapsilosis* Langeron & Talice (ATCC 22019). Cultures were obtained from the culture collection of the Phytomedicine Programme, Department of Paraclinical Sciences, Faculty of Veterinary Science, University of Pretoria. The bacteria were maintained in Mueller-Hinton (MH) agar (Oxoid, Basingstoke, UK), while the fungi were maintained in Sabouraud dextrose (SD) agar (Oxoid, Basingstoke, UK).

A 2-fold microdilution assay was used to determine the antibacterial and antifungal activities of prepared extracts [113,114]. A 100 µL sample of each was serially diluted two-fold with sterile water in wells of a 96-well microtiter plate. Subsequently, 100 µL of each bacterium or fungus adjusted to a McFarland standard of 0.5 in Mueller Hinton broth (for bacteria) or Sabouraud dextrose broth (for fungi) was added to the wells. The plates were incubated overnight at 37 °C. Regarding the fungi assay, 40 µL of *p*-iodonitrotetrazolium violet (INT, 0.2 mg/mL) (Sigma-Aldrich) was added to the plates before incubation. Gentamicin (for bacteria) and amphotericin-B (for fungi) served as positive controls, while acetone served as a negative control. For the bacteria assay, after incubation, 40 µL of *p*-iodonitrotetrazolium violet (INT) (Sigma-Aldrich) was added to the wells and incubated at 37 °C for 30 min [113].

### 3.7. Anti-Biofilm and Anti-Quorum Sensing Activity

The activity of samples on the prevention of biofilm attachment (T0) and destruction of 24 h pre-formed biofilm (T24) was assessed as previously described [115,116]. A biofilm-forming reference strain, *Staphylococcus epidermidis* (ATCC 35984), was used as the model organism for this study. Bacteria were cultured in Tryptic Soy Broth (TSB, NEOGEN, Ayr, UK) overnight before use. The biofilm was allowed to perform for either 0 h (T0) or 24 h (T24) before the addition of samples at a final concentration of 1 mg/mL. For the T0 study, standardized bacterial culture (OD_590_ = 0.02 equivalent to 1.0 × 10^6^ CFU/mL) prepared in TSB was inoculated into sterile flat-bottomed 96-well microtiter plates, followed by the addition of the extracts, and subsequently incubated for 24 h at 37 °C without shaking. For T24, cultures were pre-incubated for 24 h for biofilms to form. Then, plant extracts were added with further incubation for another 24 h. For both T0 and T24, appropriate controls included the following: negative control (culture + TSB), positive control (culture + TSB + antibiotics (gentamicin, 1 mg/mL), sample control (sample + TSB), antibiotic control (antibiotic + TSB), and media control (TSB only). After 24 h of incubation, the modified crystal violet staining (CVS) assay was done to quantify the effect on biofilm biomass [116].

Following incubation, the contents of wells were discarded, and plates were washed three times with sterile distilled water to remove unattached or loosely attached cells. The plates were dried at 60 °C for 45 min to fix the adhered biomass. Plates were then stained with 100 µL of 0.2% crystal violet solution for 30 min at room temperature. The excess stain was rinsed off by washing the plates at least five times with water. Thereafter, the biofilm biomass was evaluated semi-quantitatively by re-solubilizing the crystal violet stain bound to the adherent cells with 150 µL of 33% acetic acid to destain the wells. The absorbance of the plates was read at 590 nm using a microplate reader (Epoch™ Microplate Spectrophotometer) after careful and gentle shaking. The mean absorbance (OD_590nm_) of the sample was determined, and results were expressed as percentage inhibition using the equation below [117].

$$Percentage\;\left(\%\right)inhibition\;=\;\frac{ODNegativecontrol\;-\;ODSample}{ODNegativecontrol}\;\times \;100$$

The anti-quorum sensing activity of samples was determined as previously described [118] with minor modifications in a 48-well plate [104]. A bioreporter bacterial strain, *Chromobacterium violaceum* ATCC 12472, was used for this assay. Bacteria were cultured in Luria-Bertani (LB) broth and shaken overnight in a shaker incubator at 30 °C at 140 rpm. The bacterial suspension was diluted using LB broth to obtain a culture suspension of 5 × 10^5^ CFU/mL, which served as the working stock. Varying concentrations of plant extracts ranged from 1.25 to 0.04 mg/mL, and the inoculum was added to 48-well plates in triplicate for a final volume of 1 mL. Acetone and vanillin were included as negative and positive controls, respectively, while culture and broth controls were also included. Plates were incubated at 30 °C overnight, shaking at 140 rpm. Anti-quorum sensing activity was assessed based on the growth of the biosensor organism and the reduction of purple pigment production. The lowest extract concentration with visible growth (turbid) and no purple pigment production was interpreted as the minimum quorum sensing inhibitory concentration (MQSIC).

After incubation as described above, the plates were centrifuged at 4000 rpm for 20 min, and the supernatant was discarded. The pellets were dissolved in 1 mL of DMSO by shaking for 10 min. The plates were centrifuged again at 4000 rpm for 10 min to separate the bacteria from the solution. Then, 100 μL of the supernatant in the wells was dispensed in the wells of a 96-well microtiter plate in triplicate, and the absorbance was measured by a Biotek microplate reader at 595 nm. The percentage of violacein (purple pigment) inhibition was calculated using the below formula.

$$\%\;violaceininhibition\;=\;\frac{ODNegativecontrol\;-\;ODSample}{ODNegativecontrol}\;\times \;100$$

The concentrations of extract at which 50% of the produced violacein was inhibited (IC_50_) were calculated.

### 3.8. Cytotoxic Assessment

The cytotoxicity of extracts was tested against African green monkey kidney mammalian cells (Vero, ATCC CCL-81) using the 2,5-diphenyl-2H-tetrazolium bromide (MTT) assay [119,120]. Vero cells were maintained in a minimal essential medium (MEM, Highveld Biological, Modderfontein, South Africa) supplemented with 5% fetal calf serum (Capricon Scientific, Ebsdorfergrund, Germany) and 0.1% gentamicin (Virbac, Centurion, South Africa) in a 5% CO_2_ incubator (HERACELL 150i). Cell suspensions were prepared from 70–80% confluent monolayer cultures and plated at a density of 1 × 10^4^ in 96-well cell culture plates. Plates were incubated for 24 h at 37 °C. The cells were exposed to the extracts dissolved in the MEM at different concentrations ranging from 1 to 0.0075 mg/mL for 48 h. Doxorubicin (Accord Healthcare, Sandton, South Africa) in concentrations ranging from 0.4 to 0.005 µg/mL and dimethyl sulfoxide (DMSO) (highest concentration of 0.1%) served as positive and negative controls, respectively. The wells were thereafter rinsed with phosphate-buffered saline (PBS, Sigma-Aldrich), and 200 µL of fresh medium was dispensed into the wells. Thirty microliters (5 mg/mL) of MTT (Sigma-Aldrich) dissolved in PBS was added to each well, and plates were further incubated for 4 h at 37 °C. The medium from the wells was removed and rinsed, and 50 µL of 100% DMSO was added to the wells. Absorbance was measured immediately on a microplate reader (BioTek Synergy, Winooski, VT, USA) at a wavelength of 570 nm. Each sample concentration was tested in quadruplicate with two repeats (n = 8). The concentration causing 50% inhibition of cell viability (LC_50_) was calculated.

### 3.9. Statistical Analysis

A two-way analysis of variance (ANOVA) with post hoc Tukey’s test was used to estimate how the mean of a quantitative variable (bioactive substances) changes according to the levels of two categorical variables—solvent and plant organ (*p* ≤ 0.05 and 0.01). Pearson’s correlation coefficients were calculated to assess the interaction between bio-compounds and antioxidant capacity. Principal component analysis (PCA) was performed to evaluate how close the samples were according to the given parameters. Data were statistically analyzed using the software Statgraphics Plus 4.0 (Manugistics, Inc., Rockville, MD, USA) and SPSS 23 (IBM, Armonk, NY, USA). The visualization (Chord diagram and round tree) was generated using Flourish Studio (Canva, London, UK) and Sketch (Eindhoven, The Netherlands). The graphical abstract was generated using Biorender software (Toronto, ON, Canada).

## 4. Conclusions

The phenolic characterization of invasive species presented in this study is one step forward in the possible application areas for its rational use. The utilization of *R. pseudoacacia* as a pharmaceutical ecosystem service provider could serve as a supplementary tool in invasive species management. The enhanced knowledge on species exploitation will result in more quality monitoring of existing populations, providing additional input into IAPS management. Still, the function and significance of high phytochemical diversity are yet to be fully comprehended.

## Figures and Tables

**Table 1:** Phenolic compounds (mg/g of dry weight (DW)) of *Robinia pseudoacacia* L. leaf and flower extracts were identified by LC-MS. Values represent the mean ± standard deviation of three replicates. Different letters indicate a significant difference among the values in a row (ANOVA, Tukey’s honest significant difference, *p* ≤ 0.01); nd = not detected.

Phenolic Compounds	*Robinia pseudoacacia* Leaf		*Robinia pseudoacacia* Flower	
	70% EtOH	80% MeOH	70% EtOH	80% MeOH
3-Caffeoylquinic acid	0.454 ± 0.057 ^a^	0.355 ± 0.043 ^a^	0.123 ± 0.041 ^b^	0.037 ± 0.003 ^b^
5-Caffeoylquinic acid	0.011 ± 0.000 ^a^	0.010 ± 0.001 ^ab^	0.005 ± 0.001 ^c^	0.006 ± 0.001 ^bc^
Caffeic acid	0.346 ± 0.054 ^a^	0.236 ± 0.024 ^b^	0.100 ± 0.018 ^c^	0.059 ± 0.008 ^c^
Caffeic acid hexoside 1	0.242 ± 0.010 ^a^	0.224 ± 0.038 ^a^	0.029 ± 0.001 ^b^	0.022 ± 0.002 ^b^
Caffeic acid hexoside 2	0.039 ± 0.001 ^a^	0.021 ± 0.005 ^b^	0.003 ± 0.000 ^c^	0.004 ± 0.001 ^c^
*p*-Coumaric acid	0.152 ± 0.009 ^a^	0.116 ± 0.039 ^a^	n.d.	n.d.
*p*-Coumaric acid hexoside 1	0.052 ± 0.002 ^a^	0.048 ± 0.008 ^b^	0.007 ± 0.001 ^a^	0.006 ± 0.002 ^b^
*p*-Coumaric acid hexoside 2	0.287 ± 0.011 ^a^	0.118 ± 0.035 ^b^	0.026 ± 0.006 ^c^	0.068 ± 0.014 ^c^
3-*p*-Coumaroylquinic acid	0.102 ± 0.001 ^a^	0.086 ± 0.004 ^b^	0.001 ± 0.000 ^c^	0.002 ± 0.000 ^c^
4-*p*-Coumaroylquinic acid	0.003 ± 0.002 ^a^	0.002 ± 0.002 ^a^	0.003 ± 0.001 ^a^	0.001 ± 0.000 ^a^
5-*p*-Coumaroylquinic acid 1	0.109 ± 0.003 ^b^	0.087 ± 0.011 ^b^	0.409 ± 0.123 ^a^	0.056 ± 0.011 ^b^
5-*p*-Coumaroylquinic acid 2	0.084 ± 0.004 ^ab^	0.046 ± 0.016 ^c^	0.059 ± 0.005 ^bc^	0.101 ± 0.005 ^a^
3-Feruloylquinic acid	0.033 ± 0.001 ^a^	0.018 ± 0.004 ^b^	0.002 ± 0.000 ^c^	0.004 ± 0.000 ^c^
Ferulic acid	0.006 ± 0.000 ^a^	0.006 ± 0.000 ^a^	n.d.	n.d.
Sinapic acid derivate	0.036 ± 0.001 ^a^	0.031 ± 0.002 ^b^	n.d.	n.d.
**Hydroxycinnamic acid derivatives**	**1.956 ± 0.100 ^a^**	**1.451 ± 0.155 ^c^**	**0.603 ± 0.246 ^b^**	**0.349 ± 0.100 ^d^**
Gallic acid	0.361 ± 0.026 ^a^	0.247 ± 0.041 ^b^	0.137 ± 0.017 ^c^	0.091 ± 0.008 ^c^
Ellagic acid	0.005 ± 0.000 ^c^	0.006 ± 0.001 ^c^	0.115 ± 0.010 ^b^	0.137 ± 0.004 ^a^
Ellagic acid hexoside	1.137 ± 0.029 ^a^	0.572 ± 0.004 ^b^	0.249 ± 0.022 ^d^	0.441 ± 0.019 ^c^
Protocatehuic acid	0.192 ± 0.024	0.154 ± 0.012	0.025 ± 0.001	0.016 ± 0.001
*p*-Hydroxybenzoic acid	0.518 ± 0.013 ^b^	0.393 ± 0.070 ^a^	n.d.	n.d.
**Hydroxybenzoic acid derivatives**	**2.076 ± 0.272 ^a^**	**1.014 ± 0.751 ^b^**	**0.513 ± 0.039 ^b^**	**0.682 ± 0.028 ^b^**
Apigenin glucuronyl rhamnosylhexoside	1.036 ± 0.114 ^a^	1.220 ± 0.133 ^a^	0.051 ± 0.005 ^b^	0.091 ± 0.004 ^b^
Luteolin rhamnosyl hexoside 1	0.009 ± 0.001 ^c^	0.003 ± 0.000 ^c^	3.075 ± 0.142 ^b^	1.748 ± 0.163 ^a^
Luteolin rhamnosyl hexoside 2	0.130 ± 0.015 ^c^	0.153 ± 0.015 ^c^	1.281 ± 0.111 ^b^	1.524 ± 0.040 ^a^
Luteolin dirhamnosyl hexoside 1	0.451 ± 0.048 ^c^	0.133 ± 0.027 ^c^	6.990 ± 0.652 ^b^	12.299 ± 0.566 ^a^
Luteolin dirhamnosyl hexoside 2	0.013 ± 0.001 ^c^	0.016 ± 0.001 ^c^	1.404 ± 0.161 ^b^	2.459± 0.106 ^a^
**Flavones**	**1.52 ± 0.352 ^c^**	**1.486 ± 0.226 ^c^**	**11.474 ± 1.074 ^b^**	**19.448 ± 0.842 ^a^**
Procyanidin dimer 1	3.552 ± 0.194 ^a^	2.695 ± 0.493 ^a^	0.487 ± 0.070 ^b^	0.479 ± 0.036 ^b^
Procyanidin dimer 2	4.890 ± 0.205 ^a^	3.396 ± 0.291 ^b^	0.128 ± 0.013 ^c^	0.131 ± 0.038 ^c^
Procyanidin dimer 3	2.132 ± 0.255 ^a^	1.643 ± 0.285 ^a^	0.039 ± 0.008 ^b^	0.018 ± 0.002 ^b^
Procyanidin dimer 4	1.294 ± 0.196 ^b^	0.916 ± 0.191 ^b^	2.612 ± 0.272 ^a^	0.158 ± 0.020 ^c^
Procyanidin trimer 1	4.055 ± 0.065 ^a^	3.429 ± 0.192 ^b^	0.031 ± 0.007 ^c^	0.042 ± 0.007 ^c^
Procyanidin trimer 2	1.214 ± 0.047 ^a^	0.668 ± 0.144 ^b^	0.327 ± 0.066 ^c^	0.161 ± 0.016 ^c^
Procyanidin trimer 3	2.958 ± 0.045 ^a^	2.227 ± 0.246 ^b^	0.026 ± 0.005 ^c^	0.012 ± 0.002 ^c^
Procyanidin trimer 4	3.214 ± 0.092 ^a^	2.646 ± 0.368 ^b^	n.d.	n.d.
Procyanidin trimer 5	0.235 ± 0.028 ^a^	0.164 ± 0.046 ^b^	n.d.	n.d.
Procyanidin tetramer 1	0.728 ± 0.028 ^a^	0.401 ± 0.086 ^b^	n.d.	n.d.
Procyanidin tetramer 2	2.949 ± 0.049 ^a^	1.844 ± 0.223 ^b^	n.d.	n.d.
Epicatechin	0.013 ± 0.002 ^c^	0.009 ± 0.002 ^c^	0.251 ± 0.012 ^a^	0.105 ± 0.014 ^b^
Catechin	6.221 ± 0.194 ^a^	5.907 ± 0.493 ^a^	0.576 ± 0.081 ^b^	0.871 ± 0.141 ^b^
Gallocatechin	1.003 ± 0.043 ^a^	0.620 ± 0.077 ^b^	0.366 ± 0.081 ^c^	0.520 ± 0.054 ^c^
**Flavanols**	**32.806 ± 2.113 ^a^**	**24.357 ± 3.528 ^b^**	**4.126 ± 0.667 ^c^**	**2.134 ± 0.866 ^c^**
Quercetin pentoside	n.d.	n.d.	0.002 ± 0.000 ^a^	0.002 ± 0.000 ^a^
Quercetin-3-galactoside	0.275 ± 0.028 ^a^	0.233 ± 0.035 ^a^	0.107 ± 0.014 ^b^	0.134 ± 0.005 ^b^
Quercetin-3-glucoside	0.022 ± 0.001 ^c^	0.029 ± 0.004 ^c^	0.051 ± 0.004 ^b^	0.061 ± 0.002 ^a^
Quercetin-3-rutinoside	0.179 ± 0.020 ^b^	0.311 ± 0.047 ^b^	0.299 ± 0.085 ^b^	0.519 ± 0.033 ^a^
Quercetin rhamnosyhexoside	0.219 ± 0.047 ^b^	0.139 ± 0.021 ^b^	1.383 ± 0.160 ^a^	0.027 ± 0.002 ^b^
Kaempferol hexoside 1	n.d.	n.d.	0.003 ± 0.001 ^a^	0.004 ± 0.000 ^a^
Kaempferol hexoside 2	n.d.	n.d.	0.274 ± 0.020 ^a^	0.292 ± 0.011 ^a^
Kaempferol hexoside 3	n.d.	n.d.	0.081 ± 0.012 ^b^	0.126 ± 0.013 ^a^
Kaempferol-3-glucoside	0.200 ± 0.029 ^a^	0.170 ± 0.017 ^a^	n.d.	n.d.
Kaempferol rhamnosyl hexoside 1	0.001 ± 0.000 ^c^	0.001 ± 0.000 ^c^	0.252 ± 0.029 ^b^	0.537 ± 0.015 ^a^
Kaempferol rhamnosyl hexoside 2	0.324 ± 0.030 ^a^	0.232 ± 0.025 ^b^	0.051 ± 0.015 ^c^	0.089 ± 0.006 ^c^
Kaempferol rhamnosyl hexoside 3	0.091 ± 0.005 ^c^	0.120 ± 0.015 ^bc^	0.172 ± 0.028 ^a^	0.140 ± 0.007 ^ab^
Kaempferol glucuronyl rhamnosyl hexoside 1	0.105 ± 0.011 ^b^	0.135 ± 0.025 ^ab^	0.114 ± 0.012 ^b^	0.174 ± 0.018 ^a^
Kaempferol glucuronyl rhamnosyl hexoside 2	5.521 ± 0.623 ^a^	3.181 ± 0.182 ^b^	0.272 ± 0.033 ^c^	0.463 ± 0.041 ^c^
Isorhamnetin-3-rutinoside	1.041 ± 0.095 ^a^	1.248 ± 0.128 ^a^	0.228 ± 0.046 ^b^	0.390 ± 0.088 ^b^
Isorhamnetin hexoside 1	0.005 ± 0.000 ^b^	0.003 ± 0.000 ^b^	0.072 ± 0.025 ^a^	0.094 ± 0.004 ^a^
Isorhamnetin hexoside 2	0.093 ± 0.018 ^a^	0.058 ± 0.003 ^b^	0.010 ± 0.001 ^c^	0.017 ± 0.002 ^c^
Myricetin hexoside	0.090 ± 0.011 ^b^	0.053 ± 0.003 ^c^	0.061 ± 0.007 ^c^	0.130 ± 0.004 ^a^
Robinetin	0.001 ± 0.000 ^c^	0.002 ± 0.000 ^b^	0.003 ± 0.000 ^b^	0.003 ± 0.000 ^a^
Dihydrorobinetin	0.081 ± 0.001 ^a^	0.068 ± 0.003 ^b^	0.004 ± 0.001 ^b^	0.006 ± 0.001 ^c^
**Flavonols**	**8.226 ± 0.656 ^a^**	**5.843 ± 0.660 ^b^**	**3.457 ± 0.220 ^c^**	**3.171 ± 0.162 ^c^**
Naringenin hexoside 1	0.092 ± 0.007 ^b^	0.142 ± 0.014 ^a^	0.043 ± 0.003 ^d^	0.070 ± 0.005 ^c^
Naringenin hexoside 2	0.082 ± 0.009 ^a^	0.058 ± 0.014 ^ab^	0.057 ± 0.014 ^ab^	0.031 ± 0.010 ^b^
Naringenin hexoside 3	0.294 ± 0.039 ^a^	0.202 ± 0.020 ^b^	0.038 ± 0.008 ^c^	0.024 ± 0.005 ^c^
**Flavanones**	**0.468 ± 0.052 ^a^**	**0.362 ± 0.113 ^a^**	**0.127 ± 0.010 ^b^**	**0.125 ± 0.017 ^b^**
Vescalagin 1	3.296 ± 0.347 ^b^	1.021 ± 0.090 ^c^	3.436 ± 0.306 ^b^	5.863 ± 0.290 ^a^
Vescalagin 2	2.031 ± 0.181 ^a^	1.364 ± 0.156 ^b^	0.642 ± 0.105 ^c^	0.971 ± 0.139 ^bc^
**Ellagitannins**	**5.327 ± 0.274 ^ab^**	**1.789 ± 0.805 ^c^**	**4.078 ± 0.376 ^b^**	**6.834 ± 0.418 ^a^**
**Total Analyzed Phenolics**	**52.717 ± 2.531 ^a^**	**36.742 ± 3.588 ^b^**	**24.853 ± 2.052 ^c^**	**32.743 ± 2.2689 ^bc^**

The external standards used: caffeic acid, apigenin-7-glucoside, ferulic acid, quercetin-3-*O*-rhamnoside, neochlorogenic (3-caffeoylquinic) acid, naringenin, ellagic acid, gallic acid, chlorogenic acid, and rutin (quercetin-3-*O*-rutinoside); (-)epicatechin, quercetin-3-*O*-galactoside, quercetin-3-*O*-glucoside, *p*-coumaric acid, procyanidin B1, and kaempferol-*O*-glucoside; quercetin-3-*O*-xyloside and quercetin-3-*O*-arabinopyranoside; and isorhamnetin-3-*O*-glucoside.

**Table 2:** The yield of *Robinia pseudoacacia* flower and leaf extracts (dry extract mass obtained by the initial mass; mg/g) in different solvents; cytotoxicity (LC_50_; mg/mL) against Vero African green monkey kidney cells; minimum inhibitory concentration (MIC; mg/mL); total antimicrobial activity (TAA; yield/MIC) after 24 h of incubation (mL/g); selectivity indices (SI; LC_50_/MIC); gentamicin was used as a positive control against test bacteria and amphotericin-B against test fungi.

Plant/Organ	Solvent	Yield	LC_50_	*Pseudomonas* *aeruginosa*			*Staphylococcus* *aureus*			*Bacillus* *cereus*			*Candida* *albicans*			*Candida* *parapsilosis*		
				MIC	TAA	SI	MIC	TAA	SI	MIC	TAA	SI	MIC	TAA	SI	MIC	TAA	SI
RL	70% EtOH	204	0.11	0.63	324	0.17	>2.5	82	0.04	0.63	324	0.17	2.5	82	0.04	2.5	82	0.04
	80% MeOH	247	0.11	0.63	392	0.17	2.5	98	0.04	0.63	392	0.17	0.63	392	0.17	0.63	392	0.17
RF	70% EtOH	264	>1	>2.5	106	0.4	>2.5	105	>0.04	0.63	419	>1.59	2.5	105	0.04	1.25	211	0.8
	80% MeOH	254	>1	0.63	403	>1.59	2.5	101	0.04	0.63	403	>1.59	1.25	203	0.08	2.5	101	0.4
Gentamicin				0.0003			0.00013			0.0005								
Amphotericin-B													0.000488			0.001953		

RL—*R. psedudoacacia* leaf extract, RF—*R. pseudoacacia* flower extract; the LC_50_ of Doxorubicin, the positive control, was 0.012 mg/mL.

**Table 3:** Minimum quorum sensing inhibitory concentrations, including the half maximal inhibitory concentration (IC_50_) and antibiofilm (ABF) activities of extracts, tested on *Staphylococcus epidermidis* (for anti-quorum activity) and *Chromobacterium violaceum* (for anti-biofilm activity) models.

Plant/Organ	Solvent	Anti-Quorum Activity (mg/mL)		Anti-Biofilm Activity (%)	
		MQSIC	IC_50_	Prevention	Eradication
RL	70% EtOH	>1.25	0.43	+	+
	80% MeOH	>1.25	0.41	++	+
RF	70% EtOH	0.63	0.04	++	+
	80% MeOH	0.16	0.04	+	+

RL—*Robinia pseudoacacia* leaf extract, RF—*Robinia pseudoacacia* flower extract; (++) good ABF activity (>50% Inhibition); (+) poor ABF activity (more than 0 to 50% inhibition); (-) no ABF activity (0% or less). MQSIC = Minimum quourm sensing inhibitory concentration; IC_50_ = Half maximal inhibitory concentration.

## Data Availability

Data are presented in the manuscript.

## Supplementary Materials

The following supporting information can be downloaded at: https://www.mdpi.com/article/10.3390/plants12142715/s1, Table S1: Pearson’s correlation coefficients (two-tailed) between total phenolic (TP), total non-flavonoids (TNF) and total flavonoids (TF) contents and antioxidant capacity (obtained by DPPH, ABTS, and FRAP assays).
