# Supplementary material for: Invasive Alien Species as a Potential Source of Phytopharmaceuticals: Phenolic Composition and Antimicrobial and Cytotoxic Activity of Robinia pseudoacacia L. Leaf and Flower Extracts
Source: Plants (Basel). 2023 Jul 21;12(14):2715. doi: 10.3390/plants12142715 (PMC10385011; doi:10.3390/plants12142715)
Supplement: Supplementary file 1 [file plants-12-02715-s001.zip › plants-2464890-supplementary.pdf]

Table S1. Pearson's correlation coefficients (Two-Tailed) between total phenolic (TP), total non-flavonoids (TNF) and total flavonoids (TF) contents and antioxidant capacity obtained by DPPH (2,2-diphenyl-2-picrylhydrazyl) free radical assay, ABTS [2,2′-azino-bis (3-ethylbenzothiazoline-6-sulphonic acid)] radical cation assay, and FRAP (ferric reducing antioxidant power) assay. The results were expressed in gallic acid equivalents per g of dry weight (mgGAE/gDW) for TP and TNF and for TF in catechin equivalents per g of dry weight (mg CE/gDW).

Correlation	TNF (mg GAE/g DW)	TF (mg CE/g DW)	FRAP (mg TE/g DW)	ABTS (mg TE/g DW)	DPPH (mg TE/g DW)
TP (mg GAE/g DW)	0.331*	0.795**	0.706**	0.637**	0.782**
TNF (mg GAE/g DW)		-0.261	-0.366*	-0.375**	-0.271
TF (mg CE/g DW)			0.977**	0.937**	0.987**
FRAP (mg TE/g DW)				0.961**	0.988**
ABTS (mg TE/g DW)					0.951**

*significant correlation at the 5% level of probability ($p \leq 0.05$); ** significant correlation at the 1% level of probability ($p \leq 0.01$).
